# Monophylogenetic HIV-1C epidemic in Ethiopia is dominated by CCR5-tropic viruses–an analysis of a prospective country-wide cohort

**DOI:** 10.1186/s12879-016-2163-1

**Published:** 2017-01-06

**Authors:** Amare Worku Kalu, Nigus Fikrie Telele, Solomon Gebreselasie, Daniel Fekade, Samir Abdurahman, Gaetano Marrone, Anders Sönnerborg

**Affiliations:** 1 Division of Clinical Microbiology, Department of Laboratory Medicine, Karolinska Institute, Alfred Nobels Alle 8, F68, Huddinge, Stockholm, 14186 Sweden; 2 Department of Microbiology, Immunology and Parasitology, Addis Ababa University, Addis Ababa, Ethiopia; 3 Department of internal Medicine, Addis Ababa University, Addis Ababa, Ethiopia; 4 Public Health Agency of Sweden, Solna, Sweden; 5 Department of Science and Technology, Örebro University, Örebro, Sweden; 6 Unit of Infectious Diseases, Department of Medicine Huddinge, Karolinska Institutet, Stockholm, Sweden

**Keywords:** Subtype C, Ethiopia, Co-receptor, Antiretroviral treatment, Geno2pheno, Country-wide

## Abstract

**Background:**

CCR5 coreceptor using HIV-1 subtype C (HIV-1C) has been reported to dominate the Ethiopian epidemic. However, almost all data have been obtained from two large cities in the central and north-west regions and recent data is lacking.

**Methods:**

Plasma were obtained from 420 treatment-naïve patients recruited 2009–2011 to a large country-wide Ethiopian cohort. The V3 region was sequenced and the co-receptor tropism was predicted by the clinical and clonal models of the geno2pheno tool at different false positive rates (fpr) and for subtype. In an intention to treat analysis the impact of baseline tropism on outcome of antiretroviral therapy was evaluated.

**Results:**

V3 loop sequencing was successful in 352 (84%) patients. HIV-1C was found in 350 (99.4%) and HIV-1A in two (0.6%) patients. When comparing the geno2pheno fpr10% clonal and clinical models, 24.4% predictions were discordant. X4-virus was predicted in 17.0 and 19.0%, respectively, but the predictions were concordant in only 6%. At fpr5%, concordant X4-virus predictions were obtained in 3.1%. The proportion of X4-tropic virus (clonal fpr10%) increased from 5.6 to 17.3% (*p* < 0.001) when 387 Ethiopian V3 loop sequences dated from 1984 to 2003 were compared with ours. In an intention to treat analysis, 67.9% reached treatment success at month 6 and only 50% at month 12. Only age and not tropism predicted therapy outcome and no difference was found in CD4+ cell gain between R5-tropic and X4-tropic infected patients. At viral failure, R5 to X4 switch was rare while X4 to R5 switch occurred more frequently (month 6: *p* = 0.006; month 12: *p* = 0.078).

**Conclusion:**

The HIV-1C epidemic is monophylogenetic in all regions of Ethiopia and R5-tropic virus dominates, even in patients with advanced immunodeficiency, although the proportion of X4-tropic virus seems to have increased over the last two decades. Geno2pheno clinical and clonal prediction models show a large discrepancy at fpr10%, but not at fpr5%. Hence further studies are needed to assess the utility of genotypic tropism testing in HIV-1C. In ITT analysis only age and not tropism influenced the outcome.

## Background

Human immunodeficiency virus type 1 subtype C (HIV-1C) was isolated for the first time in 1986 from an Ethiopian patient [1] and the first near-full length HIV-1C sequence was published in 1996 [2]. By phenotypic analysis, HIV-1C isolates were found to almost exclusively use the CCR5 co-receptor for cell entry, even in patients with advanced immunodeficiency [3]. With time genotypic algorithms were developed, which enabled prediction of co-receptor usage based on V3 sequences of the envelope [4]. This approach is claimed to be applicable with a high degree of confidence also for HIV-1C, although no Ethiopian HIV-1C strains (HIV-1C_ET_) were included in these evaluations, to our knowledge [5–7].

For HIV-1 subtype B (HIV-1B), virus using the CCR5-receptor (R5-tropic) are predominant in early stages of infection, whereas virus using the CXCR4-receptor (X4-tropic) generally emerge in the advanced stages, although ultra-deep sequencing has identified that X4-variants coexist as minority strains with R5-virus in most patients [8]. Previously, X4-tropic strains have been reported to be very rare in HIV-1C infection, even in the later stage of disease [9–11]. Some recent studies from South Africa and India also showed predominance of R5 strains in treatment naïve as well as experienced HIV-1C infected patients [12], although a South African study reported predominance of X4 strains in treatment experienced children [13]. More recently, a higher incidence of X4-tropic HIV-1C were described in patients with advanced immunodeficiency from South Africa, India and Botswana, respectively [14–17]. However, recent tropism data from patients with progressive HIV-1C_ET_ infection is lacking.

In order to obtain information about the present situation in Ethiopia, we predicted the co-tropism in the largest number of HIV-1C_ET_ infected patients analysed so far. Also, since earlier reports have been derived only from the central (Addis Ababa) [18–20] and north-west (Gondar) regions [21, 22], patients from six geographical regions and from a mobile military unit were included. The aim was to get updated knowledge of whether the Ethiopian HIV-1 epidemic had become more heterogeneous and whether any regional differences exist. We also assessed the impact of baseline tropism on viral suppression and CD4+ T-cell gain after initiation of antiretroviral therapy (ART).

## Methods

### Study population

**Fig. 1:**
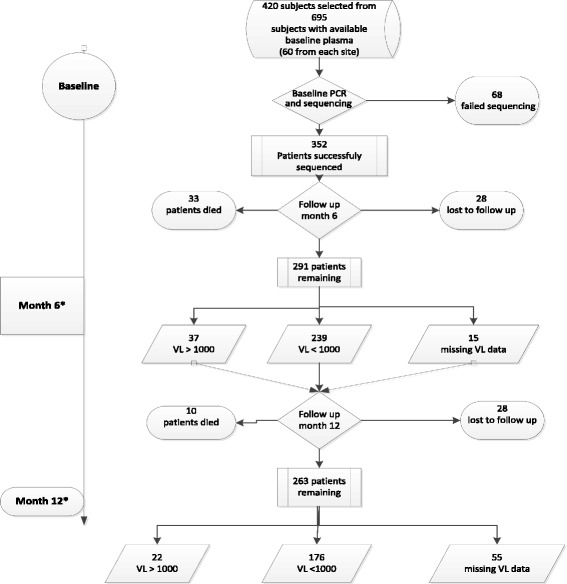
Flow chart of randomly selected patients. *Additional seven patients not included in the randomly selected group failed therapy and were included for the purpose of co-receptor switch analysis only. VL: viral load (copies/ml)

The study was conducted on 420 randomly selected ART naïve adults and adolescents (>16 years) enrolled between 2009 and 2011 in the large (*n* = 874) countrywide Ethiopian Advanced Clinical Monitoring (ACM) of HIV cohort. After inclusion to the ACM, the patients were given ART, according to the national guidelines. The patients were from clinics affiliated to medical universities in six regions (East–Harar; West–Jimma; North-west–Gondar; North–Mekele; South–Hawassa; Central–Addis Ababa) and a clinic for the Ethiopian military, the Mobile Group. From the 874 patients, baseline plasma samples were available for 695 who we stratified by study site and randomly selected 60 from each study site. Randomization was done using Microsoft Excel program (Fig. 1). The study period was 12 months and plasma was sampled at months 6 and 12. The samples were temporarily stored at −20 °C and transported thereafter to the central laboratory of the Ethiopian Health and Nutrition research institute (EHNRI) where they were stored at −80 °C. Viral load (VL) was analysed by MT 2000 real time PCR (Abbott, USA) and CD4+ T-cells were quantified by FACSCount (Becton Dickenson), at EHNRI. Ethical approval was obtained from the Ethiopian Ministry of Science and Technology and the EHNRI institutional review board. Written informed consent was obtained before inclusion.

### Database derived sequences

V3 sequences of HIV-1C_ET_ strains with a sampling date between 1984 and 2003 (*n* = 387) were downloaded from the Los Alamos database (accessed on 23th January 2015), after selecting the first sequence per patient.

### Genotypic co-receptor tropism and subtyping

HIV-1 RNA was extracted from plasma by QIAamp Viral RNA kit (Qiagen, Hilden, Germany) and cDNA was synthesized using RevertAid H-minus reagents (Life technologies, Paisley, UK), following the manufacturer’s instructions. The *env* gene encoding the V3-V4 region was amplified by nested PCR as described [23], with modified forward primer ES7 (5′ TTRTTAAATGGTAGTATAGC-3′; HXB2 nt 7001–7020) adapted for HIV-1C_ET_ [24]. The amplicons were purified by QIAquick kit (Qiagen) followed by bidirectional sequencing in automated sequencer (ABI 3130xl Genetic Analyzer, Applied Biosystems). Sequences were aligned, edited, and analysed by the BioEdit software v. 7.0.9. The V3 loop sequence was derived by gene cutter program [25] and was translated to all possible amino acid sequences as some nucleotide sequences had double peaks. Prediction of co-receptor usage was done by the clonal and the clinical Geno2Pheno algorithms as described elsewhere [4, 26], where the clinical model includes clinical data, such as nadir CD4 count and viral load, to improve prediction in treatment naïve patients. Geno2Pheno is optimized for HIV-1B but has been claimed to have 95% specificity in predicting X4-tropism in HIV-1C [7]. The false positive rate (FPR) was set to 10 and 5%, respectively, for X4 tropism and binary coded. The use of 10% FPR increases the risk of falsely predict an HIV-1 strain as R5-tropic as compared to the 5% FPR, which is of clinical relevance if the CCR5-receptor antagonist maraviroc is planned to be used. A virus was considered X4-tropic or R5-tropic, if all amino acid sequences were predicted as such. A virus that contained both kinds of amino acid sequences was classified as R5/X4-tropic [15]. Subtyping was done by the REGA subtyping tool v3.0 [27], the RIP 3.0 [24, 28], and the COMET HIV [29].

### Statistical analysis

Baseline socio-demographic and clinical characteristics (gender, age, year of HIV diagnosis, year of enrolment, CD4+ T-cells, VL) were included. The outcome of treatment was assessed with both on-treatment and intention-to treat (ITT) analysis. In the ITT analysis, ART failure was defined as either of detectable VL (>1000 copies/ml), death, lost-to-follow-up (LTFU) and missing data. Descriptive analyses included frequencies for categorical variables, and mean and standard deviation or median and interquartile range for continuous variables. Chi-square test or Fisher’s Exact Test was used to test differences between categorical variables. Independent *t*-test, Mann-Whitney, Anova and Kruskal-Wallis test assessed differences of numerical variables between two or more categories. General linear (GLM) and logistic regression models with backward selection were used for the multivariable analysis of immunological and virological responses. Beta coefficients and Odds Ratios (OR), 95% Confidence interval and *p*-values were used to present the regression models results. *P*-values < 0.05 were considered significant. Data analysis was done by the STATA software 13 (Stata Corp. College Station, USA) and IBM SPSS Statistics, version 22 (IBM Corp).

## Results

### Patient characteristics

A flow chart of the patients from baseline to month 12 is depicted in Fig. 1. Baseline V3 loop sequencing was successful in 352 of the 420 (84%) patients (Table 1). No difference in success rate was found between the sites. From baseline to month 6, 33 patients had died, 28 were lost to follow-up (LTFU) and 15 had a missing VL. Of the remaining 276 (78.4%) subjects, 37 patients had a virological failure (>1000 copies/mL; mean VL log10: 5.06; range: 3.1–7.0 copies/ml). At month 12, a further ten patients had died, 28 subjects were LTFU and 55 patients had a missing VL. Of the remaining 198 (56.3%), 22 patients had virological failure (mean VL log10: 4.9; range: 3.4–6.8 log10 copies/ml). Thus, 176 out of 352 (50.0%) reached treatment success in an ITT–analysis.

**Table 1:** Baseline characteristics of patients^a^ with a successfully sequenced V3 loop from different regions of Ethiopia

Characteristics	Overall *n* = 352	Central *Addis Ababa* *n* = 52	Mobil unit *Armed forces* *n* = 50	North-west *Gondar* *n* = 49	West *Jimma* *n* = 51	North *Mekele* *n* = 50	East *Harar* *n* = 50	South *Hawassa* *n* = 50
Gender male^b^	41.8%	38.5%	82.0%	30.6%	31.4%	32.0%	30.0%	48.0%
Age in years (mean ± SD)	34.6 ± 9.1	36.5 ± 10.8	35.2 ± 6.8	34.2 ± 8.4	32.0 ± 8.0	35.3 ± 7.8	34.4 ± 8.9	34.5 ± 11.5
Median CD4 cells/μl, IQR	120 62–185	93 38–159	91 44–175	119 75–175	132 78–220	146 64–184	131 74–196	111 68–189
Median VL log10 copies/ml, IQR	5.2 4.8–5.5	5.2 4.8–5.4	5.2 4.8–5.5	5.5 5.2–5.8	5.4 4.5–5.9	5.4 4.8–5.8	5.4 4.8–5.9	5.5 5.0–5.8

^a^A sequencing attempt was done in 420 patients; ^b^significant difference between study sites (*p* < 0.001); VL: viral load; IQR, interquartile range

### Subtyping

By the REGA tool, HIV-1C was found in 338 patients, HIV-1A1 in one patient, while no subtype was assigned for 13 patients. By RIP and COMET, the HIV-1C and HIV-1A1 classifications were confirmed. A further 12 sequences were classified as HIV-1C and one as HIV-1A1 by both methods. Thus, altogether HIV-1C was found in 350 (99.4%) of the 352 patients and A1 in two (0.6%) patients.

### Baseline tropism at fpr10% in relation to CD4+ T-cell counts and viral load

**Fig. 2:**
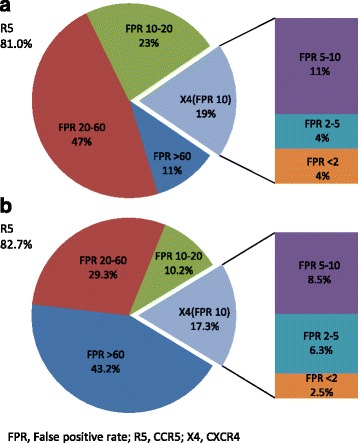
Proportions of patients with R5- or X4-tropic viruses by the geno2pheno clinical model (**a**) and by the geno2pheno clonal model (**b**). Pie plots represent: i) R5-infected (fpr >10%) patients by the fpr ranges: 10–20%, 20–60%, >60%; ii) X4-infected (fpr ≤10%) patients. Exploded bars represent the stratification of X4-infected patients according to the fpr ranges: < 2%, 2–5%, and 5–10%. FPR, False positive rate; R5, CCR5; X4, CXCR4

At baseline, 352 V3-nucleotide sequences were derived from 352 patients. These nucleotide sequences were translated into 668 amino acid sequences, which were used for tropism prediction [15] (Table 2). The patients contributed thereby with between one up to 32 amino acid sequences each. By the clinical model fpr10%, the following tropisms were predicted: R5–285 (79.0%); X4–60 (17.0%); mixed R5 and X4 (R5/X4) 7 (2.0%). At lower fpr%, the proportions of X4-virus were: fpr <2%: 4%; fpr 2–5%: 4%; fpr 5–10%: 11%) (Fig. 2a). By the clonal model, the figures were: R5–291 (82.7%); X4–50 (14.2%); R5/X4–11 (3.1%). At lower fpr%, the proportions of X4-virus were: fpr <2%: 2.5%; fpr 2–5%: 6.3%; fpr 5–10%: 8.5% (Fig. 2b).

**Table 2:** Baseline tropism predicted by the geno2pheno clinical and clonal models, respectively, at the fpr10% level

	Clinical model FPR 10%				Clonal model FPR 10%				Concordance between clinical and clonal models^a^				
	R5	X4	R5/X4	*p*-value*	R5	X4	R5/X4	*p*-value*	R5-R5	R5-X4	X4-R5	X4-X4	*p*-value**
Number (%)	285 (81)	60 (17)	7 (2)		291 (82.7)	50 (14.2)	11 (3.1)		245 (69.6)	40 (11.4)	46 (13.0)	21 (6.0)	
CD4 cells/ul median (IQR)	132 (76–193)	41 (21–98)	130 (107–220)	<0.001	125 (67–189)	89 (42–140)	160 (72–177)	0.08	135 (77–196)	127 (74–188)	55 (21–128)	70 (22–91)	<0.001
VL median (IQR)	5.3 (4.8–5.8)	5.4 (5.1–5.8)	5.3 (4.4–5.7)	0.18	5.4 (4.9–5.8)	5.4 (5–5.9)	5.4 (4.7–5.7	0.50	5.3 (4.8–5.8)	5.4 (5.1–5.9)	5.3 (4.4–5.7)	5.8 (5.3–5.9)	0.170

*Mann-Whitney *U*-Test; **Kruskal Wallis Test; ^a^R5-R5: both models predicted R5 virus; R5-X4: clinical model predicted R5 virus and clonal model predicted X4 virus; X4-R5: clinical model predicted X4 virus and clonal model predicted R5 virus; X4-X4: both models predicted X4 virus. VL: viral load (copies/ml)

Altogether, 266 (75.6%) of the 352 predictions were concordant between the two models at fpr10% (Table 2). X4-virus was present in 107 (30.4%) by any of the two models, but only 21 (6.0%) of the viruses were pure X4 by both models. A significant association was found between tropism and CD4+ T-cells by the clinical (*p* < 0.001), but not by the clonal model (Table 2). No association was found between tropism and age, gender or VL.

At the fpr5%, a 97.5% concordance was found between the two models. X4 virus was predicted by any model in 47 (13.3%) of the 352 patients. The association between tropism and CD4+ T-cells was significant by both models (*p* = 0.001; *p* = 0.023, respectively), while VL was associated with tropism by the clinical model only (*p* = 0.001).

### Co-receptor tropism in different geographical regions of Ethiopia

At fpr10%, there was no difference in occurrence of R5-virus across the geographical regions by the clinical (*p* = 0.1) (Table 3) or the clonal (*p* = 0.2, data not shown) models. At fpr5%, a significant geographical difference was observed by the clinical (*p* = 0.01), but not by the clonal model. The highest proportion of R5 tropic virus was observed in north-west Ethiopia (100%), while the lowest was observed in Southern Ethiopia (80%) (*p* = 0.001).

**Table 3:** R5-tropism across geographical regions of Ethiopia, predicted by the geno2pheno clinical model (X4 and X4/R5 combined)

Clinical model	Central *Addis Ababa n* = 52	Mobil unit *Armed Forces* *n* = 50	North-west *Gondar n* = 49	West *Jimma n* = 51	North *Mekele n* = 50	East *Harar n* = 50	South *Hawassa* *n* = 50	Total *n* = 352	*p*-value*
fpr10%	45 86.5%	41 82.0%	39 79.6%	43 84.3%	40 80.0%	44 88.0%	33 66.0%	285 81.0%	0.108
fpr5%	49 94.2%	46 92.0%	49 100%	49 96.1%	44 88.0%	47 94.0%	40 80.0%	324 92.0%	0.010

*Chi square test

### Temporal trends in X4 tropic virus among Ethiopian HIV-1 isolates

A total of 387 historical V3 loop sequences from HIV-1C_ET_ dated from 1984 to 2003 were downloaded from Los Alamos database (1984–1993: *n* = 91; 1994–2003: *n* = 296), while our study yielded 352 sequences from year 2009 to 2011. Tropism was predicted by the clonal model at fpr10% only since most historical sequences lacked clinical data. The proportion of X4/R5-X4 tropic virus increased from 5.6% (1984–1993), 7.1% (1994–2003), to 17.3% (2009–2011) (*p* < 0.001). At 5% fpr cut off, the proportion of X4/R5-X4 tropic virus increased from 1.1% (1984–1993), 3.7% (1994–2003) to 8.8% 2009–2011) (*p* = 0.003).

### Impact of baseline HIV-1 tropism on therapy response

At month 6, 291 of the 352 patients were still on ART (Fig. 1) among whom 276 (78.4%) had VL data. At bivariate analysis, no difference was seen in the proportion with plasma HIV RNA <1000 copies/mL between R5- and X4-patients on ART. In ITT analysis, by the clinical fpr10% model, X4-patients failed ART more often than R5-patients (30/67 vs 82/285; *p* = 0.013). Similar results were obtained with fpr5% (data not shown). However, multivariable analysis showed no association between tropism and outcome. In contrast, age was strongly associated with outcome at month 6 (*p* < 0.03) (Table 4), older patients with lower odds of achieving treatment success (OR = 0.922, 0.859–0.989).

**Table 4:** Multivariable analysis of baseline predictors of virological treatment response in an intention to treat analysis^a^

Follow-up	Predictor	OR (95% CI)^b^	*p*-value
Month 6	Viral tropism	1.000 (0.455–2.195)	1.000
	Age (years)	0.922 (0.859–0.989)	0.031
	Viral load	1.681 (0.915–3.090)	0.081
	CD4+ T-cell count	0.997 (0.991–1.000)	0.184
Month 12	Viral tropism	0.905 (0.248–3.302)	0.857
	Age (years)	0.920 (0.865–0.979)	0.009
	CD4 cell count	0.995 (0.989–1.001)	0.125

^a^Intention to treat analysis where treatment failure was defined as viral load >1000 copies/ml, death, LTFU or missing values; ^b^OR: Odds ratio (logistic regression model). Only variables that appeared in the final model are shown except viral tropism

At month 12, 263 were still on ART among whom 198 (56.2%) had VL data. No difference in treatment outcome was seen between patients, who had R5 or X4 virus at baseline, on ART or by ITT analysis (bivariate and multivariable). Similar results were obtained with fpr5% (data not shown). In contrast, age was strongly associated with treatment outcome at month 12 in the multivariable analysis (*p* < 0.009), older patients with lower odds of achieving viral suppression (OR 0.920; 0.865–0.979) (Table 4). It should be noted that there was no significant differences between different age groups with regard to degree of immunodeficiency when the patients were classified into three age groups (years) <30, 30–49 and >50. Thus, the median (IQR) baseline CD4+ T-cell counts were 118 (58–200), 144 (94–186) and 116 (62–181), respectively (*p* = 0.547).

At bivariate on-treatment analysis, the CD4+ T-cells was lower in X4-patients compared to R5-patients (fpr10%) at month 6 (median CD4 cell count (IQR): 200 (107–248) vs 244 (152–341) *p* = 0.017), but not at month 12. Same results were obtained for fpr5% (data not shown). However, the gain in CD4+ T-cells between baseline and month 6 did not differ between patients with R5-virus or X4-virus (fpr10%) in on treatment analysis (median, IQR: 97 cells/μl, 31–182 versus 108 cells/μl, 42–159), neither at month 12 (data not shown). Similar results were obtained when the fpr5% level was used (data not shown). In multivariable analysis, no association was found between any of the baseline predictors, including tropism, and CD4+ T-cell increase (Table 5).

**Table 5:** Multivariable analysis of baseline predictors of CD4+ T-cell increase (cells/ul)

Follow-up	Predictor	β coefficient (95% CI)^a^	*p*-value
Month 6	Viral tropism	5.8 (−42.1–53.6)	0.779
	VL log	16.6 (−1.1–34.2)	0.066
Month 12	Viral tropism	7.9 (−33.7–49.7)	0.657
	VL log	11.8 (−6.9–39.5)	0.168

^a^CD4 cell recovery difference between groups (General Linear Model, GLM). Only variables that appeared in the final model are shown except viral tropism. VL: viral load (copies/ml)

### Switch of co-receptor tropism in patients with virological failure

At month 6, virological failure was detected in 37 randomized patients and in additional seven non-randomized patients who were included only for the study of co-receptor switch. Forty-one of these patients had a plasma sample available. In their baseline samples, V3 sequencing was successful in 34 patients and R5 tropic virus was found in 29 (86.5%) of them (clonal model). Tropism switch occurred in two of 29 (7%) R5-patients and in two of five (40%) X4-patients (Fisher exact test, *p* = 0.006) at month 6. There was no difference in VL between those who switched tropism or not at baseline or at month 6 (data not shown).

At month 12, 22 subjects failed virologically in whom V3 sequencing was successful in 19 (clonal model; R5: *n* = 15; X4 or R5/X4: *n* = 4). Of these subjects, nine had also had a failure at month 6. Sixteen of the patients showed a stable tropism while the three of the four X4 or R5/X4–patients switched to R5. Of the nine patients failing both at month 6 and month 12, the tropism at month 6 was preserved at month 12 (R5: 8; X4: 1). There was no difference in viral load between patients who switched tropism or not at baseline or at month 12 (data not shown).

## Discussion

HIV-1C was first described in Ethiopia in 1986 [1] and studies have since then consistently shown that the Ethiopian epidemic is predominantly (97–100%) comprised of HIV-1C [18–22]. However, these studies have been done with very few exceptions in the larger cities of Addis Ababa (*env*) in the central region [18–20] or Gondar (*pol*) in the north-west [21, 22] and there is very limited knowledge from other parts of the country. By analysing the V3-loop of the largest number of HIV-1 strains ever sequenced from Ethiopia, we now report that HIV-1C_ET_ dominates almost exclusively all over the country. Thus, the Ethiopian HIV-1C epidemic is still monophylogenetic despite that the virus is estimated to have been introduced around 1970.

The geno2pheno tool was developed for HIV-1B, but it is used also for other subtypes, including HIV-1C [5–7]. However, in view of the unique characteristics of the HIV-1C_ET_ genome [30, 31] an evaluation of the geno2pheno for HIV-1C_ET_ is warranted. Both clonal and clinical models have a good performance when compared to phenotypic assays [4, 32]. At fpr10%, the models predicted each that around 20% of the strains were X4-tropic. However, a large discrepancy was found and only 6% were X4-tropic with both methods. Instead, when using fpr5% the concordance between the models was much higher, 97.5%, and the two models predicted concordantly X4-virus in only 3.1%. A high fpr% is valuable to decrease the risk of using the CCR5-antagonist maraviroc in patients with X4-virus, which is however not available in Ethiopia. In order to study pathogenesis a lower fpr% can be considered and we thus found that at fpr5% the Ethiopian epidemic is almost exclusively caused by CCR5-tropic HIV-1C_ET_, also in patients with very advanced immunodeficiency.

In addition to the geno2pheno tools, several genotypic tropism methods are available. A more basic approach uses the 11/25 rule, which asserts a virus as X4-tropic if amino acids at either positions 11 and 25 of the V3 loop are positively charged [33]. More sophisticated models have been developed that outperform the 11/25 rule, e.g. position specific scoring matrix (PSSM) [34] and PhenoSeq [35]. The most widely used geno2pheno and PSSM tools are highly concordant (>85%) [36]. In our study the geno2pheno tools were chosen as the European Guidelines recommend its use [6]. Moreover, since we had access to both CD4+ T-cell counts and viral load, the geno2pheno clinical tool, which has not been assessed earlier in an African setting, provided an option to potentially improve the predictions. It can be argued that there was only a 76% concordance between the clonal and clinical predictions and that a third method could have revealed this discordance. However, although a confirmatory method could have improved the prediction to some extent, we believe that none of the available genotypic methods is a preferred golden standard. Further comparison between different genotypic and phenotypic methods for the assessment of HIV-1C tropism is therefore warranted.

The CD4 cells were indeed very low (median; clinical model: 58 cells/μl; clonal model: 91 cells/μl) for X4-tropic patients, but also the R5-tropic patients had an advanced immunodeficiency (clinical model: 132 cells/μl; clonal model: 125/μl) when fpr10% was used. This is in concordance with an earlier study from Ethiopia in which patients with R5-tropic virus had a CD4 count of 78 cells/μl [37]. In contrast, no difference in viral load was found between the R5 and the X4 tropic infected patients. However, it shall be noted that the determination of co-receptor usage by a cut-off of fpr2% has been reported to be better associated with low CD4 cells and more advance disease, as compared to fpr10%, when the clonal model is used [38].

Almost all reports from Ethiopia on co-receptor usage have been done on patients from Addis Ababa or Gondar. Our study clinics were distributed all over the country and the highest prevalence of X4 tropic virus was found in southern Ethiopia (Hawassa), while the lowest was found in north-west part (Gondar) (0% versus 20%, fpr5% clinical model). There were no clinical indications or reports that patients in Hawassa got access to health care at a later stage of disease. Also CD4 cell levels and viral load were found to be independent on the geographical origin of the patients. The reasons to this difference remain to be determined and also whether the clinical outcome and the pattern of the epidemic differ in various geographical regions of Ethiopia.

At fpr10%, the bivariate analysis showed a lower rate of virologic response in X4 tropic than in R5 tropic patients at month 6 and month 12, respectively, as well as less CD4 cell increase at month 6. Earlier studies are contradictory and some authors report that X4-infected patients display poorer immunological recovery than R5-infected patients [39, 40] or similar response rates regardless of viral tropism [41]. Also, HIV-1 tropism has been reported as an independent predictor of virologic response to first-line ART with no influence on CD4 cell count recovery [42]. Differences in the methodology impede a direct comparison between these studies. Thus, it is known when using next generation sequencing almost all patients harbour X4-viruses in minor quasispecies, to varying degrees [43]. Also, the choice of fpr is an important factor, e.g. a fpr <2% has been found to be an independent factor for lower virological response and poor CD4 cell increase in HIV-1B patients [44]. Our bivariate results which showed lower virologic response in X4 infected patients both at month 6 and 12 is in agreement with these findings. However, the multivariable analysis showed higher age at the initiation of ART as the only strong independent factor associated with virologic outcome, with no difference in immunologic outcome both at month 6 and 12.

Bidirectional switch at treatment failure has been reported but without any dominance in either direction [45, 46]. However, these studies were done on HIV-1B infected patients. In our study, a switch from X4 to R5 was more common although the number of analysed samples were low. Why a viral switch during rebound seems to occur more frequently with R5-virus and if this is a phenomenon which occurs only in HIV-1C patients remains to be established.

We compared our data with historical sequence data from the Los Alamos database and found an increasing proportion of X4 virus during the last decade with the clonal fpr10% model. It is of course possible that a selection bias in the database contributed to this possible change in tropism pattern but since for most historical sequences no information about CD4+ T-cell level (or viral load) was available it was not possible to further analyse whether our patients differed clinically from those earlier reported. However, it shall be noted a similar increase in the proportion of X4 viruses has been reported from South Africa and India [14, 47].

## Conclusions

The epidemic in Ethiopia is still monophylogenetic with almost exclusively HIV-1C_ET_ strains in all geographical regions. R5-tropic strains still dominate to a high degree, but when comparing historical data, an increase of X4 viruses seems to have happened. Although the geno2pheno models are reported to have a high sensitivity in HIV-1C, our results showed a significant discrepancy in predicting X4 tropism by the clinical and clonal model at fpr10%, calling for assessment of the utility of tropism testing strategies in future studies. This is especially important in countries where the use of the co-receptor antagonist maraviroc is more common, which is not the case in Ethiopia. In contrast, a high concordance between the clinical and clonal models was seen at fpr5% giving further support to the view that R5 strains still dominate in Ethiopia.

## Abbreviations

ACM: Advanced Clinical Monitoring of HIV
ART: Antiretroviral therapy
EHNRI: Ethiopian Health and Nutrition research institute
HIV-1C: Human immunodeficiency virus type 1 subtype C
HIV-1C_ET_: Ethiopian HIV-1C strains
IQR: Interquartile range
ITT: Intention-to treat analysis
LTFU: Lost-to-follow-up
OR: Odds Ratios
R5: CCR5
VL: Viral load
X4: CXCR4
